# *Legionella pneumophila* Serotype 1 Pneumonia in Patient Receiving Adalimumab

**DOI:** 10.3201/eid1811.111505

**Published:** 2012-11

**Authors:** Terry C. Wuerz, Owen Mooney, Yoav Keynan

**Affiliations:** University of Manitoba, Winnipeg, Manitoba, Canada

**Keywords:** Legionella, Legionella pneumophila, serotype 1, lung abscess, adalimumab, anti-inflammatory agents, bacteria

## Abstract

We describe a case of severe pneumonia caused by *Legionella pneumophila* serotype 1 in a woman receiving the tumor necrosis factor–α antagonist to treat rheumatoid arthritis. As use of tumor necrosis factor–α inhibitors increase, clinicians should consider their possible association with legionellosis.

*Legionella pneumophila*, a gram-negative rod normally inhabiting aquatic environments, causes severe infections, including pneumonia (legionellosis) that can be acquired in the community or in hospitals. Traditional risk factors for legionellosis include smoking, corticosteroid use, and chronic lung disease (*1*). Receipt of a tumor necrosis factor–α antagonist (TNF-α antagonists: infliximab, adalimumab, etanercept) generally has not been considered a risk factor for legionellosis.

In the past 2 decades, use of TNF-α antagonists have revolutionized the treatment of rheumatoid arthritis, inflammatory bowel disease, psoriasis, and other inflammatory conditions. After introduction of these agents, a growing body of postmarketing literature has shown an increased risk for disease from *Mycobacterium tuberculosis*, endemic mycoses, and intracellular bacterial pathogens, including *Legionella pneumophila* (*2*). We report a severe case of right upper lobe pneumonia caused by *L. pneumophila* serotype 1, mimicking *M. tuberculosis* reactivation, in a patient receiving the TNF-α antagonist adalimumab for rheumatoid arthritis.

## Case Report

In June 2010, a 67-year-old woman sought care at an emergency department in Winnipeg, Manitoba, Canada, with a 7-day history of fevers, rigors, and progressive breathlessness. She reported diarrhea, nausea, and vomiting during the preceding 4 days. A chronic nonproductive cough was unchanged. Her medical history included rheumatoid arthritis, hypothyroidism, hypertension, and dyslipidemia. Her immunosuppressive regimen consisted of azathioprine, 150 mg per day (unchanged over the previous 2 years) and adalimumab, 40 mg per month, initiated 10 weeks earlier. She reported no history of travel or contact with persons who had tuberculosis. She denied hot tub use or other exposures to aerosolized droplets. The result of a tuberculin skin test, performed at initiation of TNF-α inhibitor, was nonreactive.

On examination, the patient appeared acutely ill. Her respiratory rate was 30 breaths per minute. Her peripheral saturation of oxygen was 96% while receiving 5 L/min oxygen by face mask. Blood pressure and heart rate were 90/60 mm Hg and 140 beats per minute, respectively; oral temperature was 38.3°C. Breath sounds were rapid with crackles noted bilaterally to the lung fields and occasional wheezes. Abdominal examination disclosed some tenderness in the right lower quadrant.

**Figure 1:**
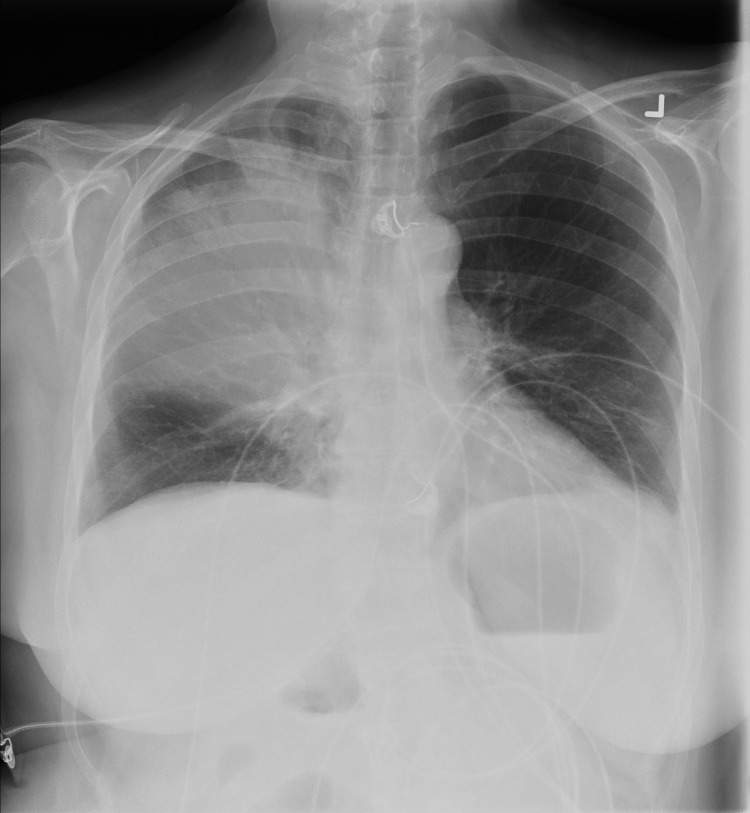
Chest radiograph demonstrating right-upper lobe consolidation in a 67-year-old woman with *Legionella pneumophila* serotype 1 pneumonia.

Laboratory investigations showed a leukocyte count of 5.9 cells/L (reference 4.5–11.0 × 10^9^ cells/L) (90% neutrophils), with toxic granulation, left shift, and Dohle bodies on the peripheral blood smear. Renal function was acutely impaired (creatinine 286 mmol/L [reference 35–97 μmol/L]); liver enzyme levels were moderately elevated (aspartate aminotransferase 150 U/L [reference 10–32 U/L], alanine aminotransferase 440 U/L [reference <25 U/L], alkaline phosphatase 75 U/L [reference 30–120 U/L]), but liver synthetic function was normal (total bilirubin 15 μmol/L [reference 3–19 mmol/L], albumin 19 g/L [reference 33–45 g/L], international normalized ratio 1.0 [reference 0.9–1.1]). An arterial blood gas suggested acidemia resulting from metabolic and respiratory acidosis with pH 7.23, pCO_2_ 41 mm Hg, HCO_3_ 17 mmol/L, and an anion gap of 23 (reference 10–12). The initial chest radiograph demonstrated right upper lobar consolidation (Figure 1).

The patient was intubated, placed on mechanical ventilation, and transferred to the intensive care unit. Her immunosuppressive agents were held, and antimicrobial drug treatment was initiated with vancomycin (because of concern about methicillin-resistant *Staphylococcus aureus* pneumonia), ceftriaxone, and azithromycin. Bronchoscopy demonstrated frank pus in the right upper lobe bronchi, cultures of which ultimately grew 2+ *L. pneumophila* serotype 1 and 2+ yeast and 1+ *Stenotrophomonas maltophilia*. Mycobacterial cultures were negative. Initial *Legionella* direct fluorescent antibody staining from the bronchoalveolar lavage was negative, as were blood cultures. A *Legionella* spp. urinary antigen test result was positive.

Antimicrobial drugs were switched on day 5 of hospitalization to levofloxacin (500 mg intravenous daily) and rifampin for *Legionella* spp. and trimethoprim–sulfamethoxisole to treat *S. maltophilia*. Over 24 hours, her clinical condition improved dramatically, leading to extubation and transfer to the general medicine ward. A computed tomographic scan of the chest, obtained 2 weeks after admission because of worsening hypoxemia, showed areas of necrosis, development of cavitary lesions within the right upper lobe, and a segmental pulmonary embolism. Anticoagulation was initiated. The antimicrobial drugs were continued for 3 weeks, then substituted for azithromycin, for a total duration of 6 weeks, which was considered an adequate duration of treatment given the cavitation. Mobilization and weaning from oxygenation were slow because of marked deconditioning and malnutrition.

**Figure 2:**
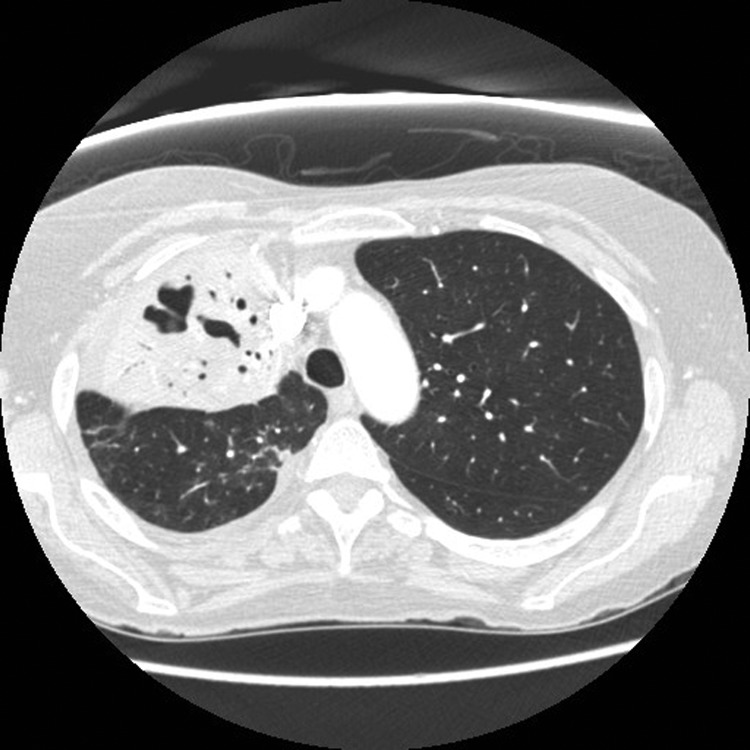
Results of repeat computed tomographic scan in a 67-year-old woman with *Legionella pneumophila* serotype 1 pneumonia 2 months after hospital admission. The scan shows reduction in the amount of consolidation and evolution of the lung cavity.

With the support of physiotherapists and other members of the integrated health care team, the patient eventually recovered and was discharged home 5 weeks after admission. Repeat computed tomographic scan 2 months after her initial illness showed evolution of the pulmonary cavity and reduced consolidation (Figure 2).

## Conclusions

Thirty-three cases of legionellosis have been described in patients receiving infliximab, adalimumab, or etanercept for rheumatoid arthritis, inflammatory bowel disease, psoriasis, or other inflammatory conditions (*3*–*5*). Similar to the case with other intracellular bacterial pathogens, such as *Listeria monocytogenes* (*2*), receipt of a TNF-α antagonist is emerging as a risk factor for pneumonia from *Legionella* spp. Lung cavitation or necrosis, which occurred in this case, is an uncommon manifestation of legionellosis and has been reported more commonly in immunocompromised hosts (*6*); we are aware of only 1 other case of lung cavitation caused by *Legionella* spp. in a patient receiving a TNF-α antagonist (*3*).

Registry data of all patients on a TNF-α antagonist in France suggested the relative risk for *L. pneumophila* infection was 16.5–21, compared with the general population (*7*). Although confounding factors, such as the effect of concomitant immunosuppressive medications or disease, are possible contributors, TNF-α antagonists themselves are likely to contribute substantially to the high risk for legionellosis in these patients. To our knowledge, azathioprine has not been associated with increased risk for Legionella infection.

Including the current report, 11 cases of *Legionella* spp. infection have been documented in patients receiving adalimumab. The researchers from France presented data indicating a higher risk for legionellosis in patients receiving infliximab or adalimumab, compared with etanercept (*8*).

The cytokine TNF-α appears to play a direct role in the immunologic response to *Legionella* spp. infection. TNF-α promotes macrophage recruitment and factors in host response to infection with intracellular pathogens (*2*). Induction of increased levels of TNF-α to macrophage cultures resulted in resistance to subsequent infection with *L. pneumophila* serotype 1; however, susceptibility was restored with addition of TNF-α antibodies to the culture (*9*). Furthermore, a mouse knockout model demonstrated 90% mortality and decreased clearance of *L. pneumophila*–infected, TNF receptor–deficient mice, in comparison with wild-type mice (*10*). *L. pneumophila* grew in TNF receptor-1 deficient macrophage culture but not when this receptor was present (*10*). The exact mechanism by which cytokine TNF-α contributes to protection from infection by *Legionella* spp. has not yet been elucidated.

Guidelines for preventing *Legionella* infection in patients receiving a TNF-α antagonist are not available; however, minimizing aerosolized exposure to untreated water sources (such as decorative fountains) is reasonable (*1*). Current data do not support avoiding the consumption of tap water.

As use of TNF-α inhibitors increase, we urge clinicians to consider this association with legionellosis. Empiric therapy for pneumonia should include an agent with activity against Legionella, such as a fluoroquinolone or macrolide.
